# A Case of Coronavirus Disease 2019 With Concomitant Acute Cerebral Infarction and Deep Vein Thrombosis

**DOI:** 10.3389/fneur.2020.00296

**Published:** 2020-04-22

**Authors:** Bo Zhou, Jianqing She, Yadan Wang, Xiancang Ma

**Affiliations:** ^1^Respiratory and Critical Care Medicine, The First Affiliated Hospital of Xi'an Jiaotong University, Xi'an, China; ^2^Cardiology Department, The First Affiliated Hospital of Xi'an Jiaotong University, Xi'an, China; ^3^Institute of Hematology, Union Hospital, Tongji Medical College, Huazhong University of Science and Technology, Wuhan, China; ^4^Department of Psychiatry, the First Affiliated Hospital of Xi'an Jiaotong University, Xi'an, China

**Keywords:** acute cerebral infarction, case, COVID-19, CT, thrombosis

## Abstract

We report a case of a 75-year-old woman diagnosed with severe coronavirus disease 2019 (COVID-19) complicated by acute cerebral infarction. The patient was admitted to our hospital on 5 February 2020 with severe COVID-19. On 20 February 2020, she was diagnosed with concomitant acute cerebral infarction *via* head computed tomography (CT) and deep vein thrombosis in both lower limbs. After symptomatic and supportive treatments, the patient was discharged on 13 March 2020. She will comply with quarantine for another 2 weeks and receive rehabilitation training from a specialist doctor. Cerebral infarction should be considered and promptly managed in patients with COVID-19.

## Background

The outbreak of coronavirus disease 2019 (COVID-19) began in early December 2019 (1, 2). Patients with COVID-19 are in a hypercoagulable state, with blood stasis and endothelial injury due to inflammation; in addition, most elderly patients with severe COVID-19 have a previous medical history of hypertension and atherosclerosis (1–4). As a result, elderly patients with COVID-19 are at high risk of thrombosis and embolism. In the present article, we report a case of a 75-year-old woman diagnosed with severe COVID-19, complicated by acute cerebral infarction and venous thrombosis of the bilateral lower extremities. The patient successfully recovered after symptomatic and supportive treatments and received rehabilitation training after discharge. Arterial and venous thrombosis could occur simultaneously and lead to poor prognosis, which should be considered and prudently managed in patients with COVID-19.

## Case Presentation

**Figure 1:**
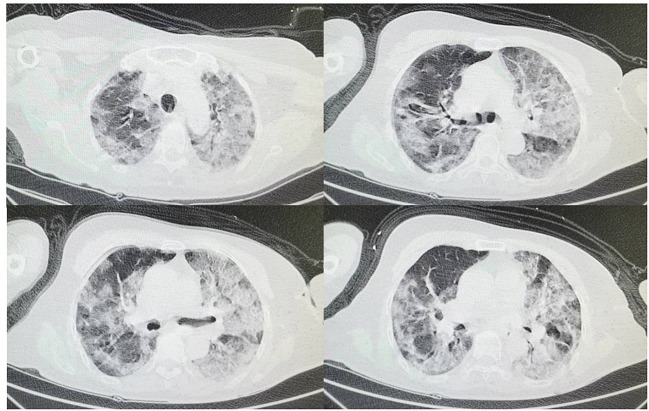
On February 5th at admission chest CT shows bilateral interstitial ground glass-like shadows and multiple consolidations.

A 75-year-old woman was admitted to our hospital on 5 February 2020 with a 2-week history of cough, fatigue, and shortness of breath without fever, hemoptysis, and diarrhea. Chest computed tomography (CT) scan showed bilateral interstitial ground glass-like shadows and multiple consolidations (Figure 1), and the nucleic acid detection of COVID-19 was positive in the local hospital before admission. Because of blood oxygen saturation of 85%, the patient was diagnosed with COVID-19 (severe) according to the New Coronavirus Pneumonia Prevention and Control Program (4th edition) published by the National Health Commission of China (5), and was transferred to the isolation ward of our hospital. Her previous medical history of hypertension for 20 years was notable.

At admission on 5 February 2020, her physical examination was normal and oxygen saturation was 95% with 3 L/min oxygen inhalation of the nasal catheter. After admission, the laboratory confirmation of COVID-19 was validated again with the Chinese Center for Disease Control and Prevention's (CDC) recommended Kit (1, 2) immediately after admission. Blood routine test showed a white blood cell (WBC) count of 5.60 × 10^9^/L (Reference: 3.5–9.5 × 10^9^/L), neutrophils percentage (NE%) of 84.7% (Reference: 40–75%), lymphocyte percentage (LY%) of 12.2% (Reference: 20–50%), hemoglobin 120 g/L (Reference: 115–150 g/L), and platelet 119 × 10^9^/L (Reference: 125–350 × 10^9^/L). The C-reactive protein (CRP) was markedly increased with the level of 42.52 mg/L (Reference: 0–8 mg/L); and the procalcitonin (PCT) was slightly increased with the level of 0.14 ng/mL (Reference: < 0.05 ng/mL). Coagulation function measurement showed that D-dimer was slightly increased with the level of 0.83 μg/mL (Reference: 0–0.5 μg/mL), and prothrombin time (PT) and activated partial thromboplastin time (APTT) was normal. Biochemical examination displayed serum creatinine (CREA) of 70 μmol/L (Reference: 57.0–111.0 μmol/L), urea nitrogen (BUN) 3.98 mmol/L (Reference: 2.9–8.2 μmol/L), alanine aminotransferase (ALT) 30 U/L (Reference: 5–40 U/L), and aspartate aminotransferase (AST) 45 U/L (Reference: 8–40 U/L). Cardiac lesion biomarkers were normal. The electrocardiogram was normal, but a cardiac/carotid artery sonography was not performed. The patient was treated immediately with arbidol (0.2 g, three times per day, po) as an antiviral agent, cefoperazone/sulbactam (2.0 g/1.0 g, three times per day, iv.drip) for antibiotic treatment, methylprednisolone (40 mg, once per day, iv) for anti-inflammatory treatment, and symptomatic and supportive treatments. On 12 February 2020 (1 week after admission), she gradually felt better, and her symptoms were gradually relieved.

When the doctor came on his daily ward round on the morning of 15 February 2020 (10 days after admission), the patient was conscious, but had limited movement (according to the manual muscle strength evaluation method: left upper limb muscle power, grade 0/5, left lower extremity muscle power, grade 0/5, right upper limb muscle power, grade 3/5, and right lower extremity muscle power, grade 3/5), inferring that the clinical diagnosis was acute cerebral infarction. We could not, however, fully evaluate facial involvement, motor coordination, muscle tonus, and sensory functions because the medical and human resources available were limited. The patient was therefore treated immediately with mannitol (50 g, once per day, iv.drip) for decreasing intracranial pressure, aspirin (100 mg, once per day, po), and clopidogrel (75 mg, once per day, po) for anti-platelet therapy.

**Figure 2:**
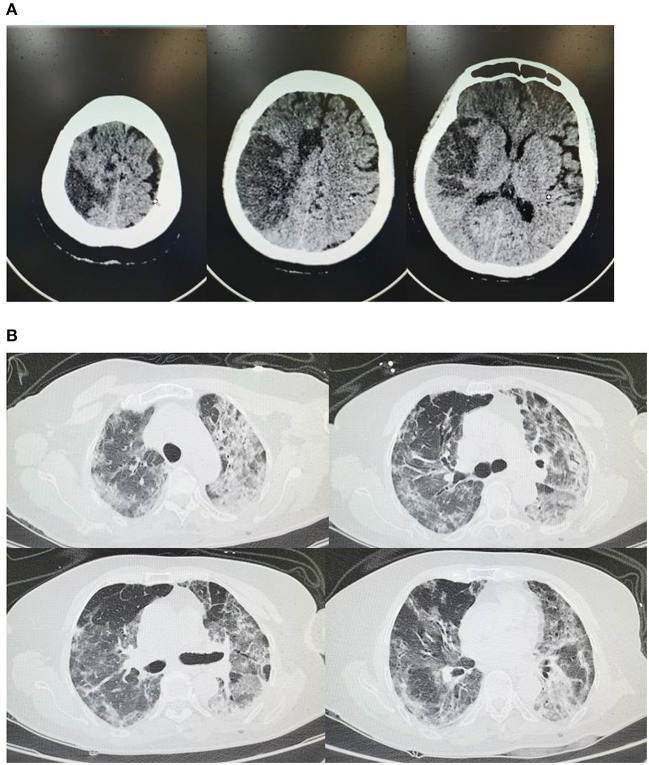
**(A)** On February 20th head CT shows bilateral cerebral infarcts involving the middle and anterior cerebral artery distribution on the right and anterior cerebral artery on the left; **(B)** On February 20th chest CT shows bilateral interstitial ground glass-like shadows and multiple consolidations.

On 20 February 2020 (2 weeks after admission), head CT scan showed bilateral cerebral infarcts involving the middle and anterior cerebral artery distribution on the right and anterior cerebral artery on the left, and chest CT scan showed bilateral interstitial ground glass-like shadows and multiple consolidations (Figures 2A,B). Rechecked blood routine test showed WBC 10.76 × 10^9^/L, neutrophils percentage (NE%) of 85.3%, lymphocyte percentage (LY%) of 9.8%, hemoglobin 106 g/L, and platelet 106 × 10^9^/L. The C-reactive protein (CRP) was slightly increased to the level of 9.39 mg/L; and the procalcitonin (PCT) was slightly increased to the level of 0.06 ng/mL. Coagulation function measurement showed that D-dimer >8 μg/mL, and PT and APTT was normal. A vascular ultrasound of the lower extremities showed extensive atherosclerosis, posterior tibial, and intermuscular venous thrombosis of the bilateral lower extremities. Rechecked urine routine test, stool routine test, liver function, renal function, and cardiac lesion biomarkers were normal. Because of relieved respiratory symptoms and decreased levels of inflammation markers, we continued treating with mannitol (50 g, once per day, iv.drip) to decrease the intracranial pressure, together with aspirin (100 mg, once per day, po) and clopidogrel (75 mg, once per day, po), and added rivaroxaban (15 mg, once per day, po) for anticoagulant treatment, and did not change the treatment for COVID-19. On 22 February 2020 (dual antiplatelet therapy for 1 week), we discontinued aspirin but continued clopidogrel (75 mg, once per day, po) and rivaroxaban (15 mg, once per day, po).

**Figure 3:**
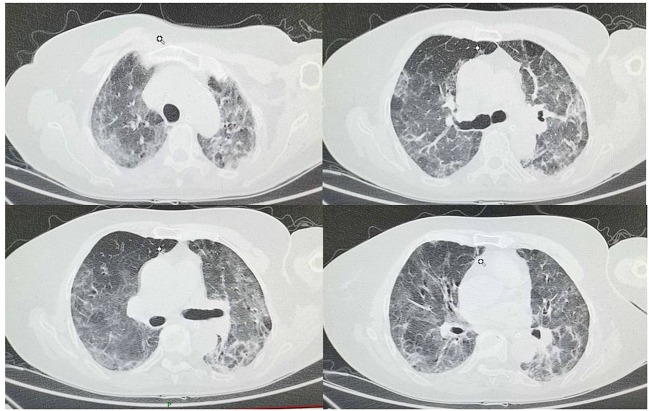
On March 4th chest CT shows less bilateral interstitial ground glass-like shadows and multiple consolidations compared to chest CT on February 20th.

On 4 March 2020 (4 weeks after admission), the patient had markedly improved and rechecked related tests were normal. Chest CT reexamination showed less bilateral interstitial ground glass-like shadows and multiple consolidations (Figure 3). Remarkably, the nucleic acid detection of COVID-19 was negative on 9 March 2020. Her respiratory symptoms were remarkably relieved, but she still had limited movement (left upper limb muscle power, grade 1/5, left lower extremity muscle power, grade 1/5, right upper limb muscle power, grade 3/5, and right lower extremity muscle power, grade 3/5). After a second nucleic acid detection of COVID-19 was negative, and the COVID-19 antibody IgM was negative, and IgG was positive, the patient was discharged on 13 March 2020. She will continue taking clopidogrel (75 mg, once per day, po) for anti-platelet therapy and rivaroxaban (15 mg, once per day, po) for anticoagulant treatment, comply with quarantine for another 2 weeks, and undergo rehabilitation training from a specialist doctor.

## Discussion

The outbreak of COVID-19 occurred in December 2019, in Wuhan, Hubei, China. It has spread rapidly around the world and has been declared a public health emergency by the World Health Organization (6–10). According to the New Coronavirus Pneumonia Prevention and Control Program (4th edition) published by the National Health Commission of China (5), severe COVID-19 has been defined as having either one of the flowing criteria: (1) Respiratory distress with a respiratory rate more than 30 times/min; (2) Oxygen saturation ≤ 93% in resting state; (3) PaO2/FiO2 ≤ 300 mmHg (1 mmHg = 0.133 kPa). Most elderly patients diagnosed with severe COVID-19 have a previous medical history with hypertension, diabetes, and atherosclerosis and so on. Additionally, a severe common bacterial and viral infection could increase the risk for blood clots. Thus, arterial and venous thrombosis should be considered and promptly managed in patients with severe COVID-19.

Acute cerebral infarction is a rare extrapulmonary manifestation of COVID-19 and few cases have been reported. The present case initially showed manifestations of severe COVID-19. While the patient was being treated for COVID-19, the sudden onset of neurological symptoms and findings of the head CT scan strongly indicated cerebral infarction in the patient. Meanwhile, our patient had normal coagulation, but showed elevated D-dimer levels, also suggesting a hypercoagulable state. However, the mechanisms underlying COVID-19-associated cerebral infarction remain unelucidated. It has been speculated that COVID-19 may disrupt the integrity of the vascular endothelium and upset the equilibrium between coagulation and anticoagulation by eliciting an inflammatory response, which may lead to hypercoagulation and thrombosis (2).

In the present case, notably, the patient had a medical history of hypertension for 20 years, and atherosclerosis was found *via* a vascular ultrasound of the lower extremities. Although the acute and progressive thrombosis might be secondary to the hyperinflammatory state during COVID-19, early anti-platelet therapy is reasonable in patients with COVID-19. Recent studies have also found that patients with severe COVID-19 usually presented with high cytokine concentrations of IL6, IL10, and TNF-α, which is associated with thrombosis and poor prognosis (2).

It has been speculated that patients with COVID-19 are prone to deep venous thrombosis (DVT). Therefore, evaluating the risk of DVT is of crucial importance in reducing the morbidity and mortality rate. High-risk DVT patients are usually over 40 years old, with more than 3 days of bed rest, and display one of the following characteristics: is over 75 years old, respiratory failure, heart failure, previous thrombosis history, acute cerebral infarction, and so on (11). In the present case, the patient had long-time hypertension, atherosclerosis, and respiratory failure; she was also in bed for more than 3 days. A vascular ultrasound of the lower extremities showed posterior tibial and intermuscular venous thrombosis of bilateral lower extremities. Therefore, for these patients, early properly antithrombotic treatment should be considered to prevent possible subsequent thrombosis events.

Moreover, the head CT showed small old cerebral infarcts on 20 February 2020, but the patient indeed did not have any limited movements prior to COVID-19 and did not have any previous medical history of stroke. Thus, we inferred that there is a possibility that the infarction was already there before admission or that it occurred right after admission but it might be difficult for physicians to have been aware of the hemiparesis during the first 10 days of treatment because the patient was too sick. Second, there might have been an old infarction in the right middle cerebral artery area without motor area involvement, but a new infarction occurred in the motor area or corticospinal tract during hospitalization. In this case the CT findings might be atypical, because the new lesion is adjacent to or surrounded by the old lesion.

A very important limitation in this case study is that we did not have arranged head CT scans for the patient, since she was hospitalized from 5 February to 20 February 2020. Additionally, the patient had limited movement on 15 February 2020, but we might have ignored early symptoms of acute cerebral infarcts before 15 February 2020, because her metabolic disturbance with hypoxia might have unmasked the motor deficit again. Thus, thrombosis could occur and lead to poor prognosis, which should be considered and prudently managed in patients with severe COVID-19.

## Conclusions

In this case report, we present one patient with severe COVID-19 and a previous history of hypertension and atherosclerosis with concomitant acute cerebral infarction, posterior tibial and intermuscular venous thrombosis of bilateral lower extremities. Our case adds further evidence of the complications of severe COVID-19. Evaluation and risk stratification for thrombosis are of vital importance for the prognosis of severe COVID-19.

## Ethics Statement

Written informed consent was obtained from the participants' next of kin for the publication of this case report, including any identifiable data or images included in this study.

## Author Contributions

BZ, XM, and YW collected the clinical and laboratory data. JS and BZ summarized the data and drafted the manuscript. BZ, XM, and YW revised the final manuscript. BZ, XM, and YW is responsible for all clinical and laboratory data.

## Conflict of Interest

The authors declare that the research was conducted in the absence of any commercial or financial relationships that could be construed as a potential conflict of interest.
